# Aortic stenosis and mitral regurgitation as predictors of atrial fibrillation during 11 years of follow-up

**DOI:** 10.1186/1471-2261-12-92

**Published:** 2012-10-18

**Authors:** Veronica Widgren, Magnus Dencker, Tord Juhlin, Pyotr Platonov, Ronnie Willenheimer

**Affiliations:** 1 Department of Cardiology, Malmö University Hospital, Malmö, Sweden; 2 Department of Clinical Sciences, Malmö University Hospital, Malmö, Sweden; 3 Department of Cardiology and Center for Integrative Electrocardiology, Lund University (CIEL), Lund, Sweden; 4 Department of Cardiology, Lund University and Heart Health Group, Malmö, Sweden

**Keywords:** Atrial fibrillation, Aortic stenosis, Mitral regurgitation, Valvular heart disease, Remodelling

## Abstract

**Background:**

There is limited information about any association between the onset of atrial fibrillation (AF) and the presence of valvular disease.

**Methods:**

We retrospectively examined 940 patients in sinus rhythm, examined by echocardiography in 1996. During 11 years of follow-up, we assessed the incidence of AF and outcome defined as valvular surgery or death, in relation to baseline valvular function. AS (aortic stenosis) severity at baseline examination was assessed using peak transaortic valve pressure gradient.

**Results:**

In univariate analysis, the risk of developing AF was related to AS (significant AS versus no significant AS; hazard ratio (HR) 3.73, 95% confidence interval (CI) 2.39-5.61, p<0.0001) and mitral regurgitation (MR) (significant MR versus no significant MR; HR 2.52, 95% CI 1.77-3.51, p<0.0001). Also the risk of valvular surgery or death was related to AS (HR 3.90, 95% CI 3.09-4.88, p<0.0001) and MR (HR 2.07, 95% CI 1.67-2.53, p<0.0001). In multivariate analyses, adjusting for sex, age, other valvular abnormalities, left ventricular ejection fraction and left atrial size − AS was independently related to both endpoints, whereas MR was not independently related to either endpoint.

**Conclusions:**

AS, but not MR, was independently predictive of development of AF and combined valvular surgery or death. In patients with combined AS and MR, the grade of AS, more than the grade of MR, determined the risk of AF and combination of valvular surgery or death. Further studies using contemporary echocardiographic quantification of aortic stenosis are warranted to confirm these retrospective data based on peak transaortic valve pressure gradient.

## Background

Valvular heart disease is a cause of atrial fibrillation (AF) [1,2]. Mitral valve disease, especially mitral regurgitation (MR), is the most common cause and the most well-known cause [3-5]. However, there is little information about the incidence of AF in patients with aortic valve disease, with or without MR.

We aimed to examine whether aortic stenosis (AS) and MR were associated with AF and long-term outcome in a large group of patients undergoing clinically motivated echocardiography examination. We designed the study to address the following hypotheses: (1) both AS and MR increase the risk of developing AF and valvular surgery or death, (2) AS in combination with MR is associated with the highest incidence of AF and valvular surgery or death, and (3) AS is at least equally important as MR for the risk of developing AF and valvular surgery or death, among individuals with combined AS and MR.

## Methods

### Study population and design

**Figure 1:**
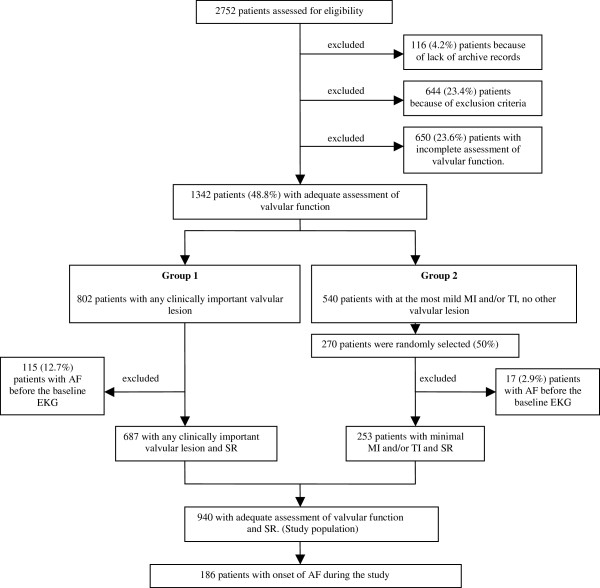
**Enrolment and outcomes.***SR*, sinus rhythm; *AF*, atrial fibrillation; *ECG*, electrocardiogram; *AS*, aortic stenosis; *AR*, aortic regurgitation; *MR*, mitral regurgitation; *TR*, tricuspid regurgitation.

All patients (n=2752) undergoing echocardiography during 1996 at the Department of Clinical Physiology, Malmö University Hospital, were assessed for possible enrolment in this retrospective cohort study (Figure 1). For patients with multiple echocardiograms in 1996, the first examination was considered as the baseline examination. The list of patients was cross- referenced with the hospital electronic patient record system, the diagnostic codes database, and the electrocardiography (ECG) database, to obtain relevant clinical information.

Exclusion criteria were: any documented episode of AF or atrial flutter before or at the time of the baseline echocardiogram, left-sided subvalvular outflow tract obstruction, congenital heart defects, aortic coarctation, and any type of valvular surgery, incomplete social security number, age less than 18 years and echocardiographic examination with poor image quality. Targeted examinations, such as control of pericardial effusion or examinations restricted to the assessment of left ventricular (LV) function only, as well as reports with incomplete echocardiographic data, were also excluded.

In addition, 650 patients with no specific report of valvular function were excluded from the study, since it could not be precluded that this might have been due to a lack of adequate assessment of valvular function. After this initial exclusion, 1342 subjects (726 women, 54%, aged 50–82 years) remained.

These patients were divided into two groups. Subjects in Group 1 had at least one valve lesion of clinical importance, defined as at least mild AS and/or aortic regurgitation (AR), and/or more than mild MR and/or tricuspid regurgitation (TR). No patient had any clinically relevant valvular lesion of any other kind. All subjects in this group (802 subjects, 430 women, 54%, aged 56–84 years) were included in the analysis. Group 2 comprised 540 patients (296 women, 55%, aged 42–77 years) with normal aortic and pulmonary valve function but with at the most mild mitral and/or tricuspid regurgitation. Half of these were randomly selected. After thorough ECG database review, a further 115 patients were excluded from Group 1 and 17 from Group 2 because of AF or atrial flutter before the baseline echocardiogram. The remaining 940 patients comprised the study population.

### Echocardiography

Patients underwent comprehensive, standardized transthoracic two-dimensional echocardiography/Doppler evaluation during 1996, by experienced echocardiography examiners, all of whom had more than 5 years experience, using Sonos 2500 equipment (Philips, Andover, Mass.). LV and left atrial (LA) dimensions were measured by 2D guided M-mode [6]. Left ventricular ejection fraction (LVEF) was assessed semi-quantitatively by 2-D visual estimation [7] and expressed in percent [8]. Valvular regurgitation was assessed semi-quantitatively by 2-D colour Doppler visual estimation [9,10] and scored from 0 through 5: 0 = no regurgitation, 1 = mild, 2 = mild-moderate, 3 = moderate, 4 = moderate-severe, 5 = severe. Grades 3–5 were considered significant. AR pressure half time was used in addition to visual assessment of AR jet size. A pressure half time of >500 ms indicated mild AR, 500–200 ms moderate AR, and <200 ms indicated severe AR. AS was evaluated quantitatively and scored as follows: 0 = no stenosis (calculated peak transaortic valve pressure gradient <20 mm Hg), 1 = mild (pressure gradient 20–30 mm Hg), 2 = mild-moderate (pressure gradient 31–40 mm Hg), 3 = moderate (pressure gradient 41–50 mm Hg), 4 = moderate-severe (pressure gradient 51–60 mm Hg), 5 = severe (pressure gradient >60 mm Hg). Grades 3–5 were considered significant. In addition, velocity time integral (VTI) ratio of the left ventricular outflow tract (LVOT) and aortic valve (AV) - (VTI LVOT/ VTI AV) and/or calculation of aortic valve area were used if LV systolic function was reduced and/or the presence of AR.

### Clinical assessment and endpoints

All patients were followed-up from the baseline echocardiogram until the date of an endpoint or until the 1st of September 2007. There were two endpoints: 1) the first registered episode of AF (occurring before valve surgery but after the baseline echocardiogram) and 2) death or the first registered episode of heart valve surgery. AF events were ascertained through thorough review of the electronic patient record systems and all ECGs recorded after the baseline echocardiogram. No distinction was made between paroxysmal, persistent or permanent AF.

### Statistical analysis

Continuous variables were expressed as mean **±** SD and between-group differences were assessed by the one-way ANOVA test. Between-group differences in categorical variables were tested with the χ2 test. Differences in time-to-endpoint were compared using the log-rank test. Between-group differences in the primary endpoints were also assessed by univariable Cox Proportional Hazards analyses initially, and then clinical (i.e. age and gender) and echocardiographic (i.e. other valvular abnormalities, LVEF and LA size) variables were included in a multivariable Cox Proportional Hazards Model with stepwise backwards selection. Although the term “other valvular abnormalities” in its true sense can mean any valvular disorder, we used it in this study to describe only the four most commonly clinically important valve conditions – AS, AR, MR and TR. As a result of the multivariable analysis, hazard ratios (HR) and 95 % confidence intervals (CI) were calculated. P<0.05 denoted statistical significance, except for the two co-primary endpoints, for which p<0.025 was considered statistically significant.

## Results

### Baseline characteristics

The study population consisted of 940 subjects (528 women, 56%) aged 51–82 years (Table 1). Compared to subjects without significant AS (n=838, 89%), subjects with significant AS (n=102, 11%) were older and more often women, and had lower LVEF and smaller LV diameter. Compared to subjects without significant MR (n=791, 84%), subjects with significant MR (n=149, 16%) were older, had lower LVEF, and larger LV and LA diameter.

**Table 1:** Baseline clinical and echocardiographic characteristics

	**All**	**No AS**	**AS**	**p-value**	**No MR**	**MR**	**p-value**
N, patients	940	838	102		791	149	
Age, years	66.6±15.4	65.6±15.5	75.2±11.4	<0.0001	65.9±15.5	70.5±14.4	0.0008
Sex (women), %	56	55	65	0.0658	57	51	0.1661
LA diameter, mm	40±7	40±7	41±6	0.0205	39±6	45±7	<0.0001
LVEF, %	54±9	55±9	49±11	<0.0001	568±8	45±12	<0.0001
LV diameter, mm	53±8	53±8	50±7	0.0062	52±7	59±10	<0.0001
AS, %	11				10	13	0.2712
MR, %	16	15	20	0.2712			
TR, %	11	10	12	0.6960	7	30	<0.0001
AR, %	11	9	29	<0.0001	10	21	<0.0001

AS-aortic stenosis grades 3–5; MR-mitral regurgitation grades 3–5; TR-tricuspid regurgitation grades 3–5; AR-aortic regurgitation grades 3–5; LA-left atrium; LV-left ventricular; LVEF-left ventricular ejection fraction.

### Univariable analysis

#### Aortic stenosis

**Figure 2:**
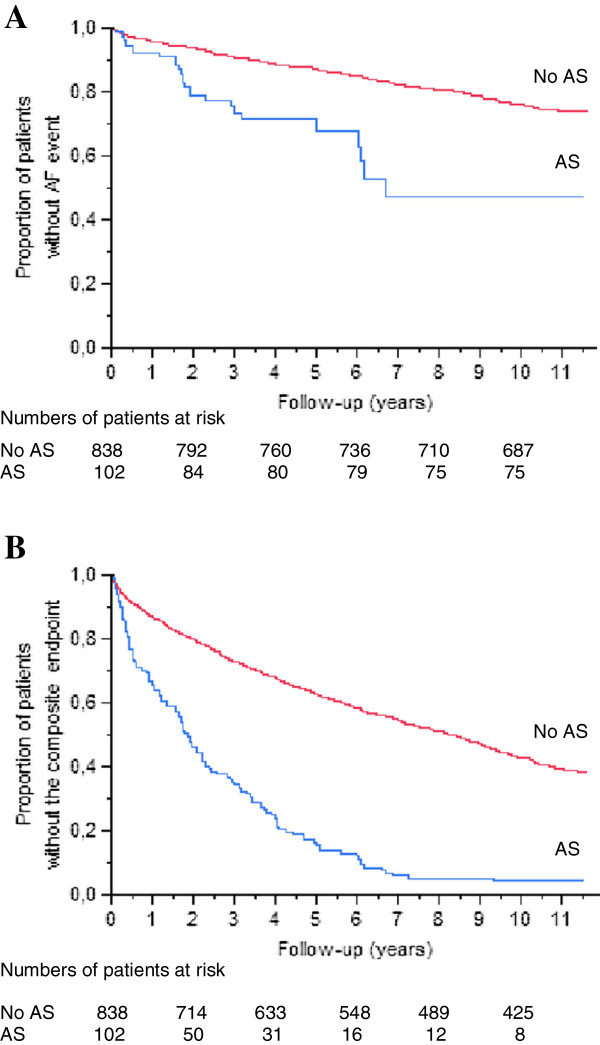
**Kaplan-Meier curves of event-free survival to AF.** (**A**) and the composite of valvular surgery or death (**B**), in patients with (blue line) and without (red line) significant AS.

More patients with significant AS experienced AF (n=27, 27%), compared to subjects without significant AS (n=159, 19%): HR 3.73, 95% CI 2.39 – 5.61, p<0.0001 (Figure 2a). More patients with significant AS experienced death or heart valve surgery (n=94, 92%), compared to subjects without significant AS (n=449, 54%): HR 3.90, 95% CI 3.09 – 4.88, p<0.0001 (Figure 2b). Median time to AF was not applicable since less than half of the patients had AF during the follow-up time. The median time to the composite endpoint was 2 years for patients with significant AS and slightly more than 10 years for subjects without significant AS (p<0.0001). Among patients with significant AS, the average annual incidence of AF was 4.1%, and for the composite endpoint the annual incidence was 14.2%. Among subjects without significant AS, the average annual incidence of AF was 2.9%, and the annual incidence for the composite endpoint was 8.2%.

#### Mitral regurgitation

**Figure 3:**
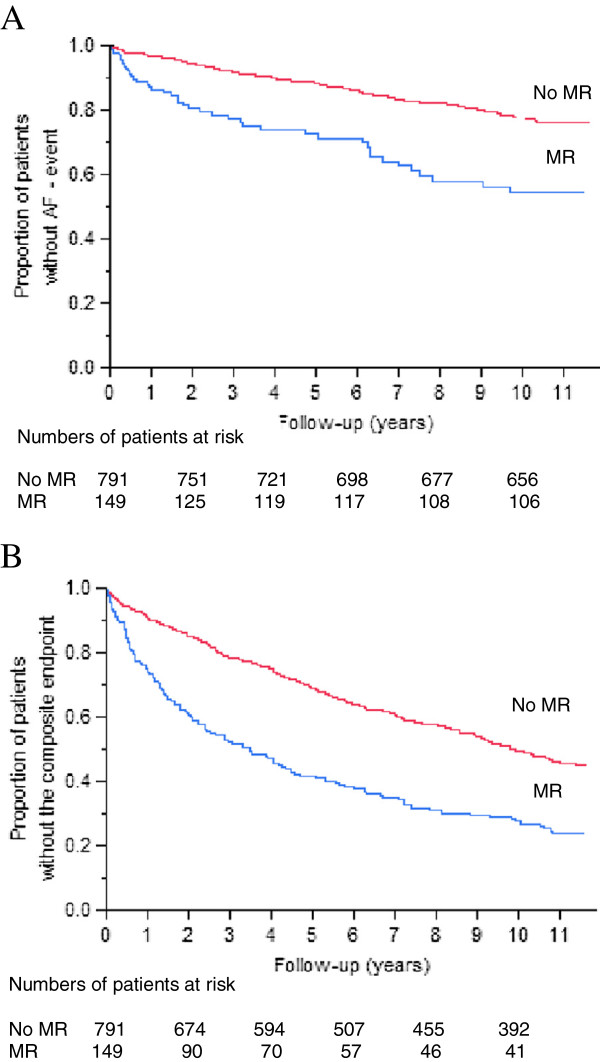
**Kaplan-Meier estimates of event-free survival to AF.** (**A**) and the composite of valvular surgery or death (**B**), in patients with (blue line) and without (red line) significant MR.

More patients with significant MR experienced AF (n=43, 29%), compared to subjects without significant MR (n=143, 18%): HR 2.52, 95% CI 1.77 – 3.51, p<0.0001 (Figure 3a). More patients with significant MR experienced the composite endpoint (n=113, 76%), compared to subjects without significant MR (n=430, 54%): HR 2.07, 95% CI 1.67 – 2.53, p<0.0001 (Figure 3b).

Median time to AF was not applicable since less than half of the patients had AF during the follow-up. The median time to the composite endpoint was 3.5 years for patients with significant MR, and 9.9 years for subjects without significant MR (p<0.0001). Among subjects with significant MR, the average annual incidence of AF was 4.4%, whereas it was 11.7% for the composite endpoint. Among subjects without significant MR, the average annual incidence of AF was 2.8%, whereas it was 8.4% for the composite endpoint.

#### Relative importance of AS and MR

We divided patients into four groups: Group A, no significant AS or MR; Group B, no significant AS but significant MR; Group C, significant AS but no significant MR; and Group D, both significant AS and MR.

**Figure 4:**
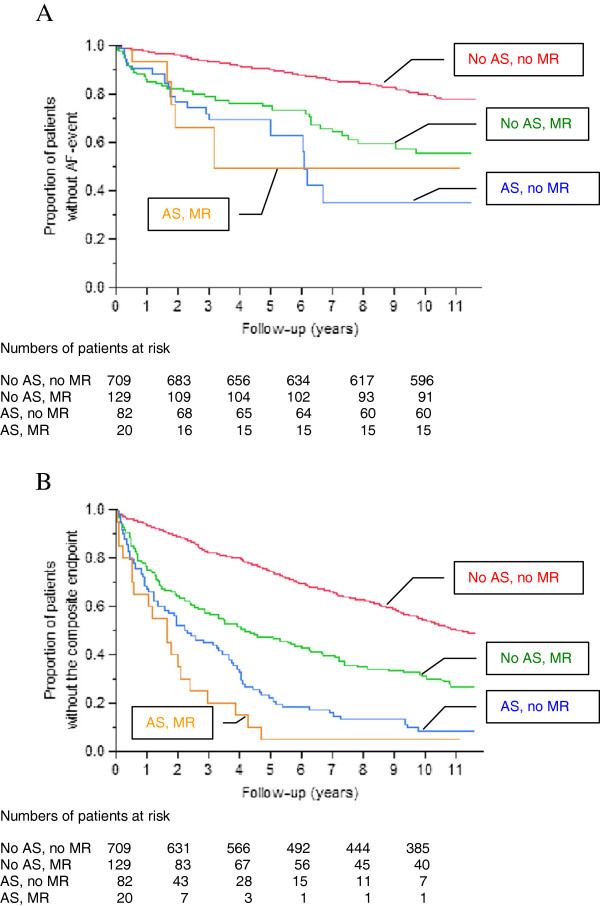
**Kaplan- Meier estimates of event-free survival to AF (A) and the composite of valvular surgery or death (B), in combined analysis**. Group A (red), Group B (green), Group C (blue) and Group D (orange).

The four-group comparison gave p<0.0001 for both AF (Figure 4a) and the composite endpoint (Figure 4b). Group D had the shortest median time to the composite endpoint (1.6 years), Group C had the second shortest (2.2 years) and Group A had the longest (11.1 years). Median time to AF was not applicable.

In order to further investigate potential differences between groups we compaired groups pairwise. Comparing Group C to Group A, HR was 4.75 (95% CI 2.89-7.49, p<0.0001) for AF and 4.28 (95% CI 3.30–5.49, p<0.0001) for the composite endpoint. Comparing Group B with Group A, HR was 2.75 (95% CI 1.88-3.92, p<0.0001) for AF and 2.15 (95% CI 1.70-2.69, p<0.0001) for the composite endpoint. Comparing Group C with Group B gave HR 1.46 (95% CI 0.83-2.50, p=0.1810) for AF and HR 1.78 (95% CI 1.30-2.41, p=0.0003) for the composite endpoint.

### Multivariable analysis

In the multivariable analysis, AS was consistently and independently related to both endpoints, whereas MR was not independently related to either endpoint (Table 2). Combined AS and MR, as a four-group variable, was independently related to both endpoints.

**Table 2:** Hazard ratios for the endpoints in relation to various risk factors in multivariate analysis

	**AF, n=186**						**Composite endpoint**					
	**AS, MR**			**AS+MR**			**AS, MR**			**AS+MR**		
	**HR**	**CI**	**p Value**	**HR**	**CI**	**p Value**	**HR**	**CI**	**p Value**	**HR**	**CI**	**p Value**
Age(year)	1.05	1.04-1.07	<0.0001	1.05	1.04-1.07	<0.0001	1.05	1.04-1.05	<0.0001	1.05	1.04-1.05	<0.0001
Sex(man)							1.30	1.09-1.55	0.0044	1.29	1.08-1.55	0.0054
AS	2.4	1.5-3.76	0.0002				2.24	1.75-2.84	<0.0001			
MR												
TR							1.44	1.11-1.84	0.0071	1.33	1.02-1.71	0.0338
AS+MR						0.0005						<0.0001
AR	0.59	0.33-0.97	0.0356	0.58	0.32-0.95	0.0306						
LVEF	0.97	0.96-0.99	0.0013	0.98	0.96-100	0.0157	0.97	0.96-0.98	<0.0001	0.98	0.97-0.99	<0.0001
LA	1.09	1.07-1.11	<0.0001	1.09	1.06-1.11	<0.0001	1.02	1.01-1.03	<0.0047	1.02	1.00-1.03	0.0177

“AS, MR” indicates that AS and MR were analysed as individual variables. “AS+MR” indicates that AS and MR were analysed as a four-group variable. AS, aortic stenosis; AR, aortic regurgitation; LA, left atrium; LVEF, left ventricular ejection fraction; MR, mitral regurgitation; TR, tricuspid regurgitation.

## Discussion

Both AS and MR were related to AF, as well as to the composite endpoint (valvular surgery or death). In the univariate analysis, patients with significant AS had 273% higher risk of AF and 290% higher risk of the composite endpoint than those without significant AS. For patients with significant MR, the risk of AF and the composite endpoint was increased by 152% and 107%, respectively, compared to patients without significant MR.

We also performed multivariate analysis with adjustment for age, sex, other valvular abnormalities, LVEF and LA diameter. Notably, AS was independently related to both endpoints, whereas MR was not independently related to either endpoint in this analysis. Age, AS, LVEF, LA and “AS+MR” as a four-group variable were independently related to both endpoints in the multivariable analysis. Sex and TR were independently related only to the composite endpoint, while there was still a significant association between AR and AF. MR was not related to either endpoint.

Our results suggest pathophysiological difference between AS and MR in AF development. It had been shown that MR causes structural changes in LA including dilatation, myofibril hypertrophy and fibrosis [11]. The atrial refractory period shortens as the atrium dilates and the vulnerability to AF increases [12,13]. LA size is a well-known powerful independent predictor of AF [14-16]. Subjects with significant MR usually have dilated LA due to volume overload. Indeed, in the present study they had on average 6.3 mm larger LA size than subjects without significant MR.

What do we know about LA size in patients with AS? Dalsgaard et al. have shown that the degree of valvular AS is an independent predictor of LA enlargement [17]. LV pressure overload causes compensatory concentric LV hypertrophy [18], which leads to both LA pressure overload and enlargement [17]. In our study individuals with significant AS only had 1.5 mm larger LA diameter than subjects without significant AS. This indicates that AF in subjects with significant AS not only precipitated by increased LA size, but rather by pressure overload with subsequent LA structural remodeling.

There is a strong and extensive evidence of atrial fibrosis being a key player in the development and persistence of AF [19,20]. Li and colleagues have shown that atrial fibrosis causes localized regions of conduction slowing, increasing conduction heterogeneity and thereby providing an AF substrate [21]. Very recently it has been demonstrated that mast cells can infiltrate the atrium of pressure-overloaded mice and contribute to the pathogenesis of atrial fibrosis and AF susceptibility [22].

AS and MR are often seen together in clinical practice. MR has been reported to be present in up to 70% of patients with symptomatic severe AS [23,24]. The present study clearly shows that, among patients with combined AS and MR, the risk for valvular surgery or death was mostly influenced by the AS. However, AS and MR were nearly equally important for the risk of AF alone. Subjects with combined AS and MR seem to have the highest risk for both AF and the composite endpoint.

The presence of AF in patients with aortic valve disease is a poor prognostic sign, associated with considerable increase in morbidity and mortality. Bergeron et al. [25] reported a 75% one-year mortality following the onset of AF in patients with AS that exceeds 50% one-year mortality following the onset of heart failure. According to the latest American [26] and European [27] guidelines for aortic valve replacement, patients with asymptomatic severe AS and preserved LV systolic function [28] should be managed by “watchful waiting” with close clinical follow-up. While these asymptomatic patients are followed-up, the pathophysiological changes in the LV lead to the structural, functional and electrophysiological abnormalities in the LA, culminating in the development of AF [29]. In patients with severe AS, the onset of AF may cause immediate clinical deterioration [18] and dramatically worsened prognosis [25]. According to current guidelines, with onset of symptoms, surgery may be considered in these patients. However, these patients are likely to have enlarged and structurally abnormal LA. AF and its hazards are difficult to control once established and, therefore, a preventive approach to the problem may be preferable [2]. Possibly, aortic valve replacement might be justified in some of these patients even at an asymptomatic stage. This approach can perhaps be more easily accepted with the development of new, less invasive methods. This should be further investigated.

### Study limitations

This study has potential limitations, besides those caused by its retrospective nature. The major limitations include the absence of some potentially important clinical patient data (e.g. history of hypertension, ischemic heart disease, hyperthyroidism or detailed medication list). We had no access to this information at the time of the baseline examination 1996, because the hospital electronic patient record system was established a few years later. Therefore, adjustment for these factors was not possible.

Due to the retrospective nature of the study, the method for AF detection in our study was based on the presence of AF on clinically motivated ECG which can obviously underestimate the true number of patients with paroxysmal form of arrhythmia and could have affected results if dedicated AF screening would had been used.

Finally, differences between echocardiography guidelines in force today and practice that existed at the time of baseline examination 1996 pose another limitation that needs to be considered while interpreting our findings. AS severity at baseline examination was assessed using mainly peak transaortic valve pressure gradient, which can be affected by other heamodynamic parameters such as the changes in cardiac output or preload state. Information on AS jet velocity, mean transaortic gradient or valve area, which are the fundametnals of AS grading today [30] was not available for all subjects and reevaluation of source historical image data not possible. In regard to LVEF, the practice at the time was to rely on visual estimation of LVEF, which may be considered not as robust as quantification techniques used today [31]. It has, however, been suggested that visual estimation of LVEF is basically as accurate as LVEF estimation by Simpson's biplane method of disc when compared to a gold standard method [8]. Also, it would have made the data more robust if only one observer had performed grading of echocardiography, findings, which however was not possible due to the nature of the investigation.

## Conclusion

During 11 years of follow-up of elderly patients, AS, but not MR, was independently related to AF and combined valvular surgery or death, irrespective of adjustment for age, sex, other valvular disease, LVEF and LA diameter. In addition, AS was more important than MR for the risk of combined valvular surgery or death, whereas AS and MR were about equally important for the risk of developing AF. To the best of our knowledge, this is the first study assessing the importance of AS and combined lesions of AS and MR to the development of AF. However, further studies using contemporary echocardiographic quantification of aortic stenosis are warranted to confirm these retrospective data based on peak transaortic valve pressure gradient.

## Competing interests

The authors declare that they have no competing interests.

## Authors’ contributions

The authors’ contributions were as follows: VW, MD, TJ and RB designed the study. VW collected data. VW, PP and RB carried out the data analysis. VW, PP and RB drafted the manuscript, and all authors made critical revisions of the manuscript. All authors read and approved the final manuscript.

## Pre-publication history

The pre-publication history for this paper can be accessed here:

http://www.biomedcentral.com/1471-2261/12/92/prepub
